# A case report of synchronous triple primary malignancies: Diffuse large B-cell lymphoma, rectal adenocarcinoma and hepatocellular carcinoma

**DOI:** 10.3389/fonc.2022.1046878

**Published:** 2022-12-21

**Authors:** Beixuan Qiu, Cheng Lin, Lupeng Wu, Yifei Li

**Affiliations:** ^1^ Department of XingLin General Surgery, First Affiliated Hospital of Xiamen University, Xiamen, China; ^2^ Department of Hematology, First Affiliated Hospital of Xiamen University, Xiamen, China; ^3^ Department of Hepatobiliary & Pancreatovascular Surgery, First Affiliated Hospital of Xiamen University, Xiamen, China

**Keywords:** multiple primary malignancies, diffuse large B-cell lymphoma, rectal cancer, hepatocellular carcinoma, diagnosis, treatment

## Abstract

A 59-year-old man was admitted to our hospital in August 2020 because of fever with night sweats and weight loss. The patient was eventually diagnosed with synchronous triple primary malignancies: diffuse large B-cell lymphoma (DLBCL), rectal adenocarcinoma and hepatocellular carcinoma (HCC), which has not been reported previously. The patient initially received six cycles of R-Gemox chemotherapy targeting DLBCL, the response to the treatment was partial remission. We continued six cycles of R-CHOP therapy, and DLBCL achieved a complete remission to treatment. During R-CHOP chemotherapy, PD-1 inhibitor (Sintilimab) was used to control the disease progression of HCC, which was effective and tolerable. Subsequently, he successfully completed curative intent Dixon operation and right hemihepatectomy. The diagnosis and treatment for like these synchronous triple primary malignancies are a huge challenge, herein we provide our experience in this regard.

## Introduction

Warren and Gates first defined multiple primary malignancies (MPM) as two or more histopathologically distinct malignancies in the same individual. MPM are classified as synchronous when tumors are diagnosed within 6 months of each other, otherwise as metachronous (1). Metachronous presentation is more frequent than synchronous, with a ratio of about 2-4 (2, 3). MPM mainly involve breast, lung, prostate, and melanoma. Synchronous lymphoma and solid digestive system tumor are a relatively rare scenario (3). Cases of patients with coexistence of 2 malignancies, such as colorectal cancer with diffuse large B- cell lymphoma (DLBCL), hepatocellular carcinoma (HCC) with DLBCL, rectal cancer with HCC, have been reported (4, 5). However, to our knowledge, there is no reported case of synchronous triple primary malignancies involving rectal cancer, HCC and DLBCL. Here we report for the first time an extremely unusual case with these 3 malignancies coexisting in the same patient. No diagnosis and treatment standards for this type of case have been established. This study aims to provide our experience on one case in this regard.

## Case presentation

A 59-year-old man was admitted to the general surgery department of the First Affiliated Hospital of Xiamen University in August 2020, due to fever to 38.5°C, accompanied with night sweats and weight loss of 7kg over the past 1 month. The patient had no personal and family history of malignant neoplasm. An abdominal contrast-enhanced computed tomography (CT) scan taken at another hospital a few days before admission revealed the following: a space-occupying lesion of the rectal; one diameter 15-mm hypervascular lesion with arterial phase enhancement followed by portal venous phase washout in couinaud segment 8 (S8) of liver; multiple low-density shadows scattered in the liver parenchyma; and multiple hypovascular nodules in spleen.

After admission, routine laboratory test results showed that lactate dehydrogenase: 1331 u/L (normal range, 120-250 u/mL), albumen was 29 g/L (normal range, 40-55 g/L), and C-reactive protein was 76.8 ng/L (normal range, 0-6 ng/L). The virological examination indicated the patient was infected with hepatitis B virus (HBV). The remaining parameters including tumor markers were in the normal range. Positron emission tomography-computed tomography (PET-CT) exposed multiple abnormal fluorodeoxyglucose (FDG) uptake in rectum (standardised uptake value (SUV)max 6.7), liver (SUVmax 6.4), spleen (SUVmax 5.5), bone (SUVmax 12.0), left parotid gland (SUV max 21.2) and multiple enlarged lymph nodes in the bilateral cervical and periclavicular, right hilar, mediastinal, retroperitoneal, para-aortic, bilateral internal and external iliac regions (SUVmax 20.7) (**Figuress1A, E**). However, PET-CT showed that liver S8 had no metabolic uptake, which had an enhanced lesion in previous CT. From the above B-symptoms and radiological findings, the patient was considered to have a hematological disease involved multiple organs, and then was transferred to the Department of Hematology in our hospital for further examinations and treatments.

**Figure 1:**
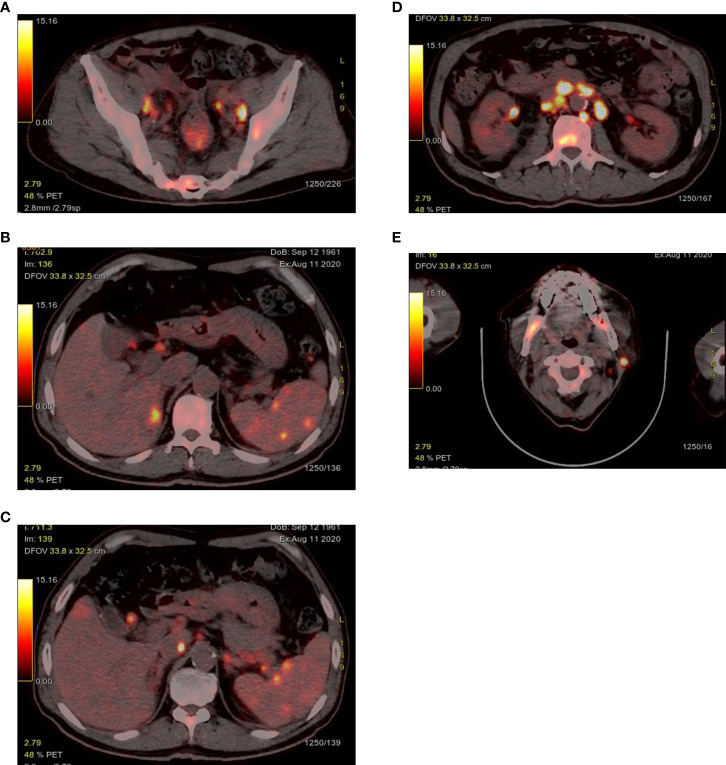
PET-CT exposed abnormal FDG uptake in the rectum **(A)**, liver and spleen **(B, C)**, bone **(A, D, E)**, left parotid gland **(E)** and multiple enlarged lymph nodes.

**Figure 2:**
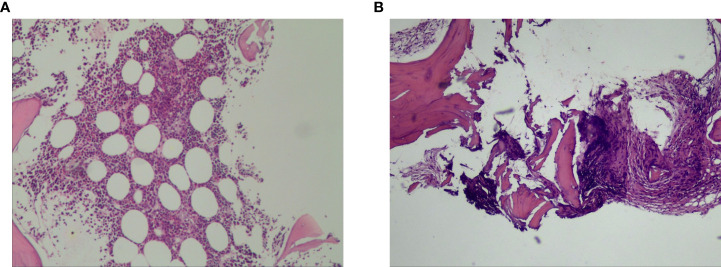
Histopathological examination of biopsies from both the retroperitoneal enlarged lymph nodes and liver lesions revealed diffuse large B- cell lymphoma **(A)**. Bone marrow biopsy of L4 vertebrae was positive for lymphoma **(B)**.

**Figure 3:**
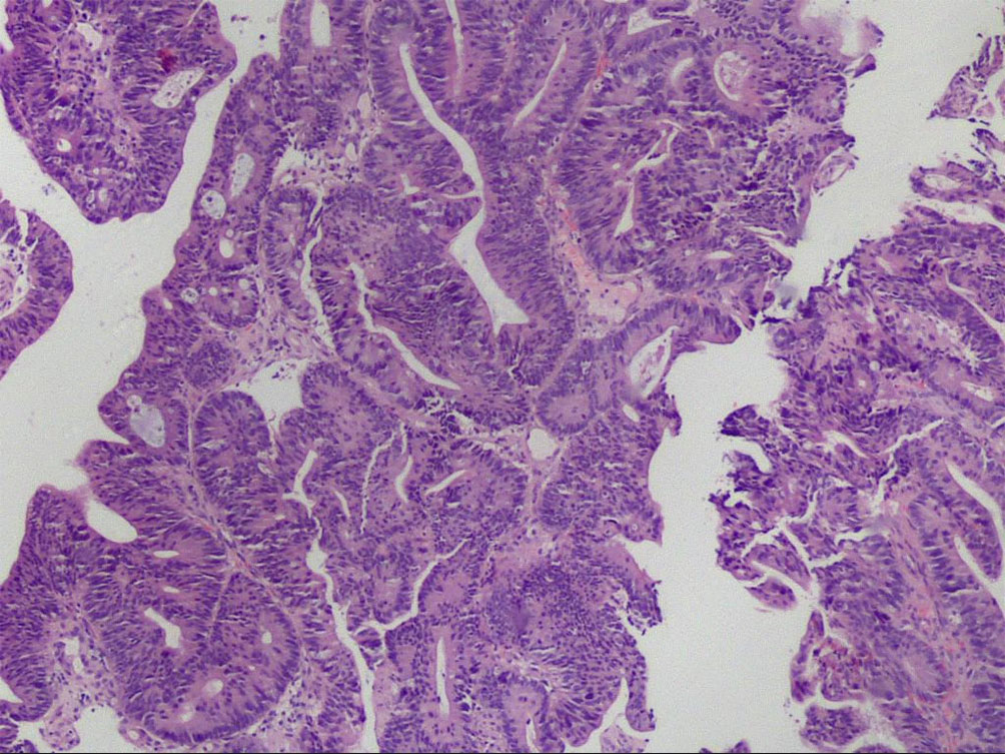
The colonoscopic biopsy suggested moderately differentiated adenocarcinoma.

PET-CT guided percutaneous needle biopsies of retroperitoneal enlarged lymph nodes and Hepatic S6 nodule were performed. Histopathological examination of biopsies from both the retroperitoneal enlarged lymph nodes and liver lesions revealed DLBCL (**Figure 2A**), with CD20(+), CD30(30%+), Bcl-2(+), Bcl-6(+), MUM-1(+), c-Myc(30–60%+), P53(40%+). The Ki67 proliferation index was 80%. *In situ* hybridization showed EBER was negative. Bone marrow biopsy of L4 vertebrae was positive for lymphoma (**Figure 2B**). In order to exclude other primary diseases, further colonoscopy and biopsy were performed. The colonoscopy showed a hard-intraluminal ulcerated mass in the rectum, approximately 8cm from the anal verge, and the biopsy of the lesion suggested moderately differentiated adenocarcinoma (**Figure 3**). Based on pathology and PET scan findings, the patient was diagnosed with synchronous primary stage IV DLBCL and rectal adenocarcinoma.

**Figure 4:**
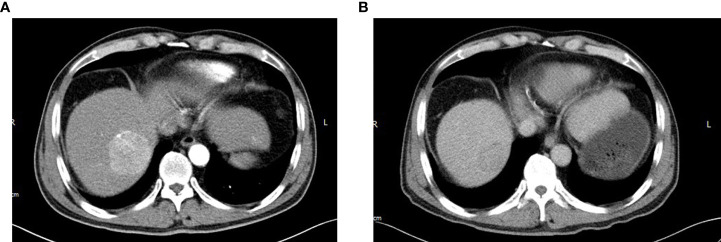
After six cycles of R-Gemox chemotherapy, follow-up abdominal enhancement CT exposed that the hepatic S8 lesion has a rapid progression measuring longest diameter 51mm with arterial phase enhancement **(A)** with portal venous phase washout **(B)**.

**Figure 5:**
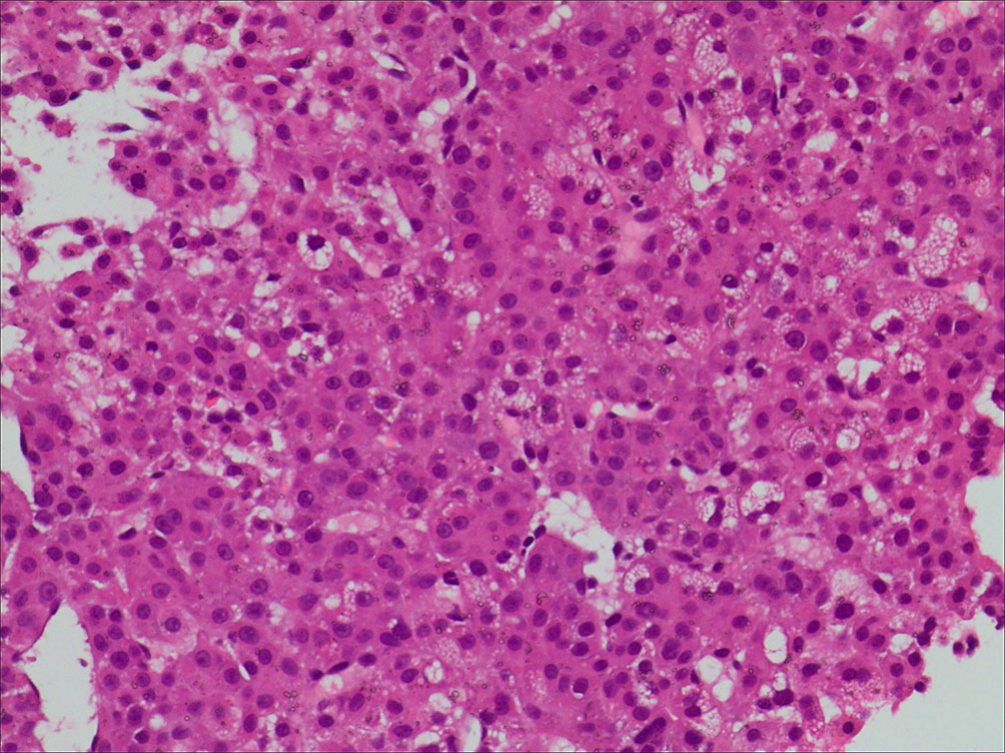
Pathological examination of liver needle biopsy confirmed that the S8 lesion was hepatocellular carcinoma.

After considering the tumor burden of both diseases and the performance status of the patient, it was decided to initially treat the stage IV DLBCL given that the lymphoma was more likely to affect survival than the asymptomatic rectal cancer. He received six cycles of chemotherapy with R-Gemox (rituximab, gemcitabine, oxaliplatin), and was good tolerance. An interim PET-CT showed that the majority of enlarged lymph nodes and bone metastases with high uptake of FDG disappeared, and the rest significantly shrinked. There was no metabolic uptake in the liver, spleen and left parotid gland. PET-CT revealed a slight rise uptake value in the rectum (SUVmax 7.9). In all, the response to DLBCL treatment was assessed as partial remission (PR), and the rectal cancer was relatively stable. However, follow-up abdominal enhancement CT exposed that the hepatic S8 lesion showed in previous CT scan has a rapid progression measuring longest diameter 51mm with imaging characteristics of “quick wash-in and wash-out” (**Figures 4A, B**), which strongly suggested HCC. Liver needle biopsy confirmed that the S8 lesion was HCC (**Figure 5**). The diagnosis time interval between DLBCL and HCC was less than 5 months. Finally, the patient was diagnosed with synchronous triple primary malignancies: DLBCL, rectal adenocarcinoma and HCC.

A multidisciplinary team of hematologist, oncologists, general surgeon and hepatobiliary surgeon proposed a combination therapy plan, comprising R-CHOP (rituximab, cyclophosphamide, doxorubicin, vincristine and prednisolone) targeting DLBCL plus PD-1 inhibitor sintilimab (200mg every 3 weeks) targeting HCC. After six cycles of combined therapy, follow-up PET-CT found that DLBCL was complete remission (CR) to the therapy this time, and abdominal CT showed a reduction of more than 20% of the HCC mass, which was evaluated as PR.

With the cure of DLBCL and relief of HCC, laparoscopic Dixon operation was performed to treat rectal cancer in June 2021. The post-operative course was uneventful. The patient was discharged 6 days after the operation. Pathological examination confirmed a moderately differentiated adenocarcinoma, with tumor-free circumferential and distal margins, without lymph nodes metastases (pT3N0M0, stage IIA). Immunohistochemistry revealed Ki-67(80%+), MLH1(+), MSH2(+), PMS2(+), MSH6(+). Postoperative adjuvant chemotherapy was considered unnecessary.

One month later, enhanced magnetic resonance imaging (MRI) detected the second progression of S8 HCC with a size of 74 mm, and a diameter 20mm new tumor was found in the S6 of liver, which was considered an intrahepatic metastasis of HCC. Then, radical right hemihepatectomy was performed in August 2021, and postoperative histological analysis of specimens from both S8 and S6 of the liver was compatible with HCC. Some four months after resection, MRI detected local recurrence, and he underwent percutaneous microwave ablation (MVA). The process was mundane and the patient was dismissed one day after the operation. Two months after MVA, contrast-enhanced ultrasound showed that the recurrent tumor was completely inactivated. She remains alive and well without evidence of any tumor recurrence till now. (The medical process is shown in **Table 1**).

**Table 1:** The timeline of diagnosis and treatment process.

Date	Medical process	Result
July 8, 2020	Onset of disease	Fever, night sweats and weight loss
August 8, 2020	Abdominal enhancement CT	A space-occupying lesion of the rectal; one diameter 15-mm hypervascular lesion with arterial phase enhancement followed by portal venous phase washout in couinaud segment 8 (S8) of liver; multiple low-density shadows scattered in the liver parenchyma; and multiple hypovascular nodules in spleen.
August 10, 2020	Routine blood tests	Lactate dehydrogenase: 1331 u/L (normal range, 120-250 u/mL), Albumen: 29 g/L (normal range, 40-55 g/L) C-reactive protein: 76.8 ng/L (normal range, 0-6 ng/L) HBsAg: positive
August 12, 2020	PET-CT	Multiple abnormal FDG uptake in rectum, spleen, bone, left parotid gland and multiple enlarged lymph nodes in the bilateral cervical and periclavicular, right hilar, mediastinal, retroperitoneal, para-aortic, bilateral internal and external iliac regions. Image diagnosis: Lymphoma
August 13, 2020	Transfer to	Hematology department
August 14, 2020	Biopsies	Retroperitoneal enlarged lymph nodes, hepatic S6 nodule and bone marrow: histopathological examination confirmed DLBCL
August 16, 2020	Colonoscopy	A hard-intraluminal ulcerated mass in the rectum, approximately 8cm from the anal verge, and the biopsy suggested moderately differentiated adenocarcinoma
August 22, 2020	Diagnosis	1.DLBCL(Stage IV) 2.Rectal adenocarcinoma
August 25, 2020 to January 22, 2021	Chemotherapy	R-Gemox (rituximab, gemcitabine, oxaliplatin)
January 28, 2021	PET-CT and abdominal enhancement CT	DLBCL : Partial remission, Rectal adenocarcinoma:Disease stability Abdominal enhancement CT: Hepatic S8 lesion has a rapid progression measuring longest diameter 51mm with imaging characteristics of “quick wash-in and wash-out”
January 30, 2021	Liver needle biopsy	Histopathological examination confirmed that the S8 lesion was HCC
February 5, 2021	Updated diagnosis	1.DLBCL(Stage IV) 2.Rectal adenocarcinoma 3. HCC
February 7, 2021	Multidisciplinary conference	Multidisciplinary team proposed a combination therapy plan, comprising R-CHOP (rituximab, cyclophosphamide, doxorubicin, vincristine and prednisolone) targeting DLBCL plus PD-1 inhibitor sintilimab (200mg every 3 weeks) targeting HCC.
February 8, 2021 to June 22, 2021	Combination therapy	R-CHOP+ PD-1 inhibitor
June 26, 2021	PET-CT and abdominal enhancement CT	DLBCL : Complete remission, Rectal adenocarcinoma:Disease stability, HCC::Partial remission
June 29, 2021	Surgery	Laparoscopic Dixon operation
July 22, 2021	Abdominal enhancement MRI	Progression of S8 HCC with a size of 74 mm, and a diameter 20mm new tumor was found in the S6 of liver
August 18, 2021	Surgery	Radical right hemihepatectomy
December 22, 2021	Follow-up MRI	Local recurrence of surgical margin
December 28, 2021	Ablation	Percutaneous microwave ablation
August 10, 2022	Last follow-up	Alive with disease-free

## Discussion

Synchronous MPM refer to two or more tumors occurring within 6 months of each other. The following three criterias have been proposed by Warren and Gates to characterise MPM, (i) each tumour must be distinct from the other; (ii) each must have well-defined malignancy characteristics; (iii) the probability that one is a metastasis derived from the other must be excluded (1). Our case met all these criteria. Therefore, we can state that the patient in this report suffered from synchronous MPM consisting of three tumors. The previously similar reported instances of synchronous triple primary malignancies including lymphoma and solid digestive system tumors are listed in **Table 2** (6–8).

**Table 2:** The previously reported examples of synchronous triple primary malignancies including lymphoma and solid digestive system tumors.

Author	Year	Age/Gender	MPM	Treatment	Prognosis
Chong (06)	2010	80/M	DLBCL	No treatment	Died 30 days after diagnosis
			HCC		
			GA		
Wang (07)	2019	78/M	DLBCL	Radical resection	Died of stroke about 1year after diagnosis
			SA	Radical resection	
			MDS	4 cycles of chemotherapies	
Dayer (08)	2021	67/F	DLBCL	R- CHOP chemotherapy	Alive after 1- year follow- up
			RA	Rectal endoscopic excision of the colorectal tumour	
			PNT	Laparoscopic pancreatectomy	
Present case	2022	59/M	DLBCL	R-GEMOX and R-CHOP chemotherapy	Alive 2 years after diagnosis
			RA	Radical resection	
			HCC	Sintilimab targeted therapy right hemihepatectomy microwave ablation	

Aetiological factors with MPM may include genetic predisposition and family cancer syndromes, immunosuppression, immunodeficiencies and infection, hormonal factors, environmental and lifestyle exposures, carcinogenic effects of prior cancer treatments (9). Andersen et al. suggested that chronic HBV infection was associated with all-type cancer, but not non-Hodgkin’s lymphoma (10). Therefore, HBV infection may contribute to some extent to the development of synchronous rectal cancer and HCC in our case. Michele et al. reported that among MPM with HCC, B cell neoplasms are associated with HCC more frequently than other cancers, and at a higher incidence than in the general population (11). Besides, a partial Bccip defect is found to be a risk factor for spontaneous hepatocellular carcinoma and B-lymphoma development (12). These findings attempted to explain the phenomenon of the coexistence of HCC and DLBCL. With regards to colorectal tumor and lymphoma, Barron and Localio suggested that patients with lymphoma have an increased incidence of synchronous colorectal carcinoma, and lymphoma may be the initial event which suppresses the patient’s defenses against the development of colorectal carcinoma (13). In addition, Hirano et al. observed a case that both colon cancer and lymphoma showed microsatellite DNA instability, sharing alteration in a locus of chromosome 7 (D7S501) (14). Nonetheless, the rarity of such cases prevents any firm conclusion regarding the pathophysiology of the relationship between these 3 coexistent malignancies of rectal cancer, HCC, DLBCL.

Diagnosis of synchronous MPM may be difficult and one of these malignancies could be missed by the practitioner in some clinical situations. Such missed cases have been reported. A 61-year-old male was diagnosed with follicular lymphoma by an excision biopsy of cervical enlarged lymph node. PET-CT showed focal pathological uptake in the stomach, which was considered an infiltration of lymphoma into the stomach. After two cycles of R-CHOP chemotherapy, PET-CT indicated residual FDG uptake in the stomach. Subsequently, gastroscopic biopsy was performed. Astonishingly, histopathology revealed gastric tubular adenocarcinoma, and no infiltration of lymphoma to the stomach was found (05). Risio et al. reported a similar case of postoperative pathologically confirmed DLBCL of the colon with synchronous liver metastasis, which was considered preoperatively to be metastatic colorectal adenocarcinoma due to no liver biopsy (15). Therefore, clinicians should be aware of the possibility of MPM, whenever a patient with multiple lesions distributed in different organs. Biopsy is necessary for suspicious lesions, especially multiple lesions of different organs, because preoperative diagnosis can be challenging to the radiologist. During the initial diagnostic work-up in our case, abdominal CT revealed multiple low-density foci without enhancement in the liver, and these lesions showed hypermetabolism in the PET-CT. After a liver biopsy, it was confirmed that these low-density foci were DLBCL. Meanwhile CT images revealed one 1.5cm hypervascular space-occupying lesion with “quick wash-in and wash-out” in S8 of the liver, which has obvious heterogeneity compared with other liver DLBCL lesions. However, since PET-CT revealed no abnormal uptaken radioactivity in the S8 lesion, the liver biopsy was not performed on this lesion before the treatment of DLBCL, leading to that HCC was not immediately diagnosed. These clinical observations highlight the need for more focus on the investigation of the diagnosis for MPM. Multidisciplinary collaboration between the different specialists should be performed for careful investigations and accurate diagnosis.

Treatment protocols for MPM are not well established, even more so is the treatment for the synchronous presentation of rectal cancer, HCC, DLBCL. Colorectal cancer is the third most frequently diagnosed cancer and the second most lethal cancer worldwide (16). Treatments for early rectal cancer include curative intent colectomy, neoadjuvant therapy (17). Primary liver cancer ranks sixth in terms of incidence, but third in terms of mortality in the world (16). For HCC, the common treatments include surgical resection, ablation, transarterial chemoembolization, target immunotherapy (18). DLBCL is the most common subtype of non-Hodgkin’s lymphoma. It is one of the aggressive lymphomas that can be cured, even in advanced cases. R-CHOP chemotherapy is the most appropriate treatment (19). When our patient was diagnosed with synchronous rectal cancer and DLBCL, curative intent colectomy was infeasible at that time, thus the systemic treatment regimens must consist of drugs targeting these two different kinds of tumors. Gemcitabine plus oxaliplatin (Gemox) has shown significant antitumor activity in solid digestive system tumors (20). On the other hand, rituximab plus gemcitabine and oxaliplatin (R-Gemox) is highly effective in non-Hodgkin’s lymphoma (21). So, we chose R-Gemox as initial therapy, which was effective and tolerable. However, during R-Gemox chemotherapy, the HCC had a rapid progression with the longest diameter from 15 to 51mm. Surgical resection is the gold standard for treatment of primary liver cancer. However, the patient was not candidates for hepatic resection because of aggressive lymphoma. Microwave ablation (MVA) is an effective treatment for patients with early-stage HCC, especially tumor smaller than 20mm, which is comparable with hepatic resection. And percutaneous MVA has become a recognized treatment approach because of its efficacy, reproducibility, low complication rates, and availability (22). If we provided early detection and timely accurate diagnosis when the diameter of S8 lesion was 15mm, and MVA was performed in the initial treatment modality, the patient may have a better outcome, even avoiding right hemihepatectomy. Therefore, successful treatment of MPM required a good multidisciplinary collaboration to provide timely correct diagnosis and the best therapeutic strategy based on the diagnosis.

In summary, this is an unusual presentation of multiple primary malignancies of rectal adenocarcinoma, DLBCL and HCC. The study of this case may provide useful information regarding the diagnosis and treatment of patients with similar conditions. We concluded the following points (1): Clinicians must keep in mind that patients with multi-organ simultaneous lesions may suffer from MPM (2). We suggest a scrupulous biopsy of suspicious lesions in patient with multiple lesions of different organs to improve the diagnostic accuracy (3). The standard diagnosis and treatment protocols for MPM remain unclear. To handle these complex cases, require a multidisciplinary team with expertise and effective teamwork.

## Data availability statement

The original contributions presented in the study are included in the article/supplementary material. Further inquiries can be directed to the corresponding author.

## Ethics statement

Written informed consent was obtained from the individual(s) for the publication of any potentially identifiable images or data included in this article.

## Author contributions

BQ and YL contributed equally to this article. BQ wrote the first draft of the manuscript. BQ and CL collected the data. YL and LW revised the manuscript. YL contributed to conception and design of the study and designed the manuscript. All authors contributed to the article and approved the submitted version.
